# Saponin accumulation in the seedling root of *Panax notoginseng*

**DOI:** 10.1186/1749-8546-6-5

**Published:** 2011-01-24

**Authors:** Dong Wang, Hongtao Zhu, Keke Chen, Min Xu, Yingjun Zhang, Chongren Yang

**Affiliations:** 1 State Key Laboratory of Phytochemistry and Plant Resources of West China, Kunming Institute of Botany, Chinese Academy of Sciences, Kunming 650204, PR China

## Abstract

**Background:**

*Panax notoginseng *is an important Chinese medicinal plant. Dammarene-type triterpenoid saponins are main pharmacologically effective compounds in *P. notoginseng*. This study aims to investigate the formation and accumulation of saponins in *P. notoginseng *roots during germination and juvenile stage.

**Methods:**

*P. notoginseng *seeds were collected and stored in wet sand. After germination, the seedlings were transplanted into a soil nursery bed and cultivated for one year. During this period, samples were collected every month and the concentrations of ginsengnosides Rg_1_, Re, Rb_1_, Rd and notoginsengnoside R_1 _were determined by HPLC.

**Results:**

There was little saponin in the *P. notoginseng *seed. The chemical composition of seed was different from that of root. After germination, Rb_1_, Rg_1_, Re, Rd and R_1 _appeared successively in the seedling root. And in the five-month-old root, all these five main saponins came into existence. The accumulation of saponins in *P. notoginseng *root was affected by seasons.

**Conclusion:**

The accumulation of saponins showed a time-dependent increase after germination of *P. notoginseng*.

## Background

*Panax notoginseng *(Burk.) F. H. Chen. (*Sanqi*), a species belonging to the Araliaceae family, is an important medicinal plant for its haemostatic and restorative properties [1]. Much chemical and pharmacological research on *P. notoginseng *has been carried out, indicating that dammarene-type triterpenoid saponins are not only the main chemical components but also the main pharmacologically effective components. They exert various effects on the cardiocerebral vascular system, central nervous system and endocrine system [2-7]. To date, over 70 dammarene-type triterpenoid saponins have been isolated from the whole plant of *P. notoginseng *[8-10], with the major saponins isolated from the root being ginsenosides Rg_1 _(Rg_1_), Re (Re), Rb_1 _(Rb_1_), Rd (Rd) and notoginsenoside R_1 _(R_1_).

Although detailed chemical studies have been carried out on mature root and leaf of *P. notoginseng*, very little information is available on the chemical composition of its seed or on saponin accumulation during root development. The present study aims to investigate the formation and accumulation of saponin constituents in *P. notoginseng *during germination and juvenile stages, particularly the developmental changes of Rg_1_, Re, Rb_1_, Rd and R_1 _in the seed and seedling root.

## Methods

### Solvents and chemicals

Methanol (MeOH) was purchased from Tianjing chemical Ltd (China). Acetonitrile (MeCN) was purchased from Merck (Germany). Standard Rg_1_, Rb_1_, Re, Rd and R_1 _were isolated from the root of *P. notoginseng *and their chemical structures were determined with nuclear magnetic resonance (NMR) and mass spectrometry (MS).

### Plant materials

Mature seeds were collected from a 3-year-old *P. notoginseng *in November 2005 from the farm of Miaoxiang Ltd (Wenshan County, Yunnan Province, China). After removal of the pericarp, the seeds were stored in wet sand; after germination, the seedlings were transplanted into a soil nursery bed under a sheltering net with 80% shadowiness. During this period, 50 g seed or 20 individual seedling roots were sampled at intervals of one month until seedlings grew to 12 months old. The samples were dried at 45°C and powdered. Concentrations of Rg_1_, Re, Rb_1_, Rd and R_1 _were analyzed with a high-performance liquid chromatography (HPLC).

### Seed extraction

After removal of the pericarp, 100 g of fresh seeds was crushed and extracted at room temperature with MeOH (300 ml) for three times. The concentrated MeOH extract was partitioned between water and petroleum ether. The aqueous part was concentrated under reduced pressure as a crude seed extract (0.3 g).

### HPLC analysis

A Waters Alliance HPLC (USA) equipped with Alliance separation module 2695 and photodiode array detector 2996 was used in the analysis. A reversed-phase column (Waters Symmetry C-18, 3.9 × 150 mm i.d., 5 μm) was used. The gradient elution system consisted of water (A) and acetonitrile (B). Separation was achieved using the following gradient: 0-20 min: 20%-22% B, 20-45 min: 22%-46% B, 45-55 min: 46%-55%, 55-60 min: 55%-90% B. The column temperature was set at 25°C. The flow rate was 1 ml/min. The UV detection wavelength was 203 nm. The mean values of three replicates were calculated.

### Method validation

The method was validated by measuring Rg_1_, Re, Rb_1_, Rd and R_1_. Instrument precision was obtained by analyzing the peak areas of six injections. The relative standard deviations (RSDs) were: R_1_: 1.09%, Rg_1_: 0.97%, Re: 1.39%, Rb_1_: 1.04% and Rd: 1.03%. Stability of sample solution was measured by injecting the same sample solution at time points of 0, 6, 12 and 24 hour. The RSDs were: R_1_: 0.96%, Rg_1_: 0.43%, Re: 1.35%, Rb_1_: 0.54% and Rd: 1.41%. Reproducibility was evaluated by measuring the concentrations of these five analytes in six replicate samples with external standards. The RSDs were: R_1_: 0.80%, Rg_1_: 0.36%, Re: 1.24%, Rb_1_: 0.58% and Rd: 0.50%. The recovery rates of these five saponins were determined by the method of standard addition with six replications. The average recovery rates and the RSDs were calculated (Table 1).

**Table 1:** Recovery rates of five main saponins in *P. notogingseng*

Saponin	Spiked (mg)	Saponin detected mean (SD) (mg)	Recovery rate (%)	RSD (%)
R_1_	2.68	2.74 (0.07)	102.2	2.44
Rg_1_	18.91	20.12 (0.15)	106.4	0.85
Re	0.86	0.86 (0.02)	100.0	2.38
Rb_1_	10.60	11.27 (0.02)	106.3	1.81
Rd	2.12	2.08 (0.04)	98.1	1.49

Recovery rate (%) = (mean of amount detected/spiked amount) × 100%

RSD: relative standard deviation

### Standard curve

Standard solutions were prepared by combining Rg_1 _(0.332 mg/ml), Rb_1 _(0.344 mg/ml) as standard solutions A whereas Re (0.047 mg/ml), Rd (0.082 mg/ml), R_1 _(0.084 mg/ml) as solution B; both were dissolved in methanol. The standard curves were generated by injecting standard solutions of 5 μl and 10 μl to 90 μl at the intervals of 10 μl respectively. The peak area for each analyte was determined. Standard curves were then constructed (Table 2).

**Table 2:** Regression equations of five main saponins in *P. notoginseng*

Saponin	Regression equation	*P*	***r***^**2**^	Test range (μg)	LOD (ng)	LOQ (ng)
R_1_	*y *= 584809*x*-47740	<0.001	0.9999	0.42-7.56	1.05	2.80
Rg_1_	*y *= 645054*x*+46049	<0.001	0.9999	1.66-29.88	1.11	2.77
Re	*y *= 616766*x*-36027	<0.001	0.9993	0.24-4.23	1.18	2.65
Rb_1_	*y *= 456796*x*+125610	<0.001	0.9996	1.72-30.96	1.15	2.46
Rd	*y *= 614439*x*-14119	<0.001	0.9999	0.41-7.38	1.02	2.73

*y*: peak area;

*x*: amount of analyte (μg);

LOD: S/N = 4

LOQ: S/N = 10

*r*^2^: coefficient of determination

S/N: signal to noise ratio

### Limits of detection and limits of quantitation

The standard solutions were diluted with 70% aqueous methanol to provide appropriate concentrations. When the ratio of the testing peak signal-to-noise (S/N) was 4, the limit of detection (LOD) for each analyte was determined; when the S/N ratio was 10, the limit of quantitation (LOQ) was determined.

### Sample preparation for HPLC analysis

For seed samples of germination test, 1.0 g of powder was weighed accurately and extracted ultrasonically for 30 minutes in 70% methanol in a 10 ml volumetric flask. After cooled down and made up the lost volume with methanol, the sample solution was obtained by filtering the supernate with a nylon filter membrane (0.45 μm) prior to the HPLC analysis. For seedling root samples, 50 mg of powder was weighed accurately and extracted in 70% methanol ultrasonically in a 5 ml volumetric flask. The other steps were similar to those of the seed sample. Injection volumes of seed and seedling sample solutions for HPLC were 100 μl. As to HPLC analysis of seed extract, the raw extract was dissolved in MeOH (10 mg/ml) and filtered with 0.45 μm nylon filter membrane and 10 μl of solution was injected for HPLC analysis.

### Statistical analysis

Linear regression was performed with Excel 2003 (Microsoft, USA). RSDs were also calculated with Excel 2003 (Microsoft, USA).

## Results

Under the HPLC conditions used in this study, all five saponins were baseline separated and their calibration curves exhibited good linear regressions. The method validation analysis demonstrated that the analytical method developed in this study for all five saponins was accurate and precise.

The *P. notoginseng *seeds were collected in November in our experiments; peeled seeds were stored in wet sand which started to germinate in the following January. Seedlings were then transplanted into a soil nursery bed in February and they would grow till the third February. Samples were collected every month, the contents of ginsenosides Rg_1_, Re, Rb_1_, Rd, notoginsenoside R_1 _were analysed with HPLC. The RSDs of the concentrations of five analytes of each sample in triplicate analyses were all within 3% (Table 3).

**Table 3:** Saponin concentrations (%) in seed and seedling root of *P. notoginseng *during seed germination and juvenile stage

	Month	**R**_**1**_	**Rg**_**1**_	Re	**Rb**_**1**_	Rd
Seed	Nov	ND	ND-	ND-	ND-	ND-
	Dec	ND	ND-	ND-	ND-	ND-
	Jan	ND	ND-	ND-	ND-	ND-
						
Seedling root	Feb	ND	ND-	ND-	0.002	ND-
	Mar	ND	ND-	ND-	0.061	ND-
	Apr	ND	ND-	ND-	0.059	ND-
	May	ND	0.046	0.028	0.092	0.002
	Jun	0.036	0.122	0.029	0.098	0.017
	Jul	0.056	0.232	0.036	0.106	0.018
	Aug	0.062	0.322	0.049	0.240	0.050
	Sep	0.168	0.664	0.077	0.405	0.107
	Oct	0.214	0.552	0.095	0.627	0.122
	Nov	0.178	0.566	0.111	0.439	0.074
	Dec	0.158	0.541	0.092	0.431	0.078
	Jan	0.146	0.375	0.063	0.380	0.053
	Feb	0.109	0.447	0.051	0.309	0.040

ND: not detected

### Saponins in the seed

**Figure 1:**
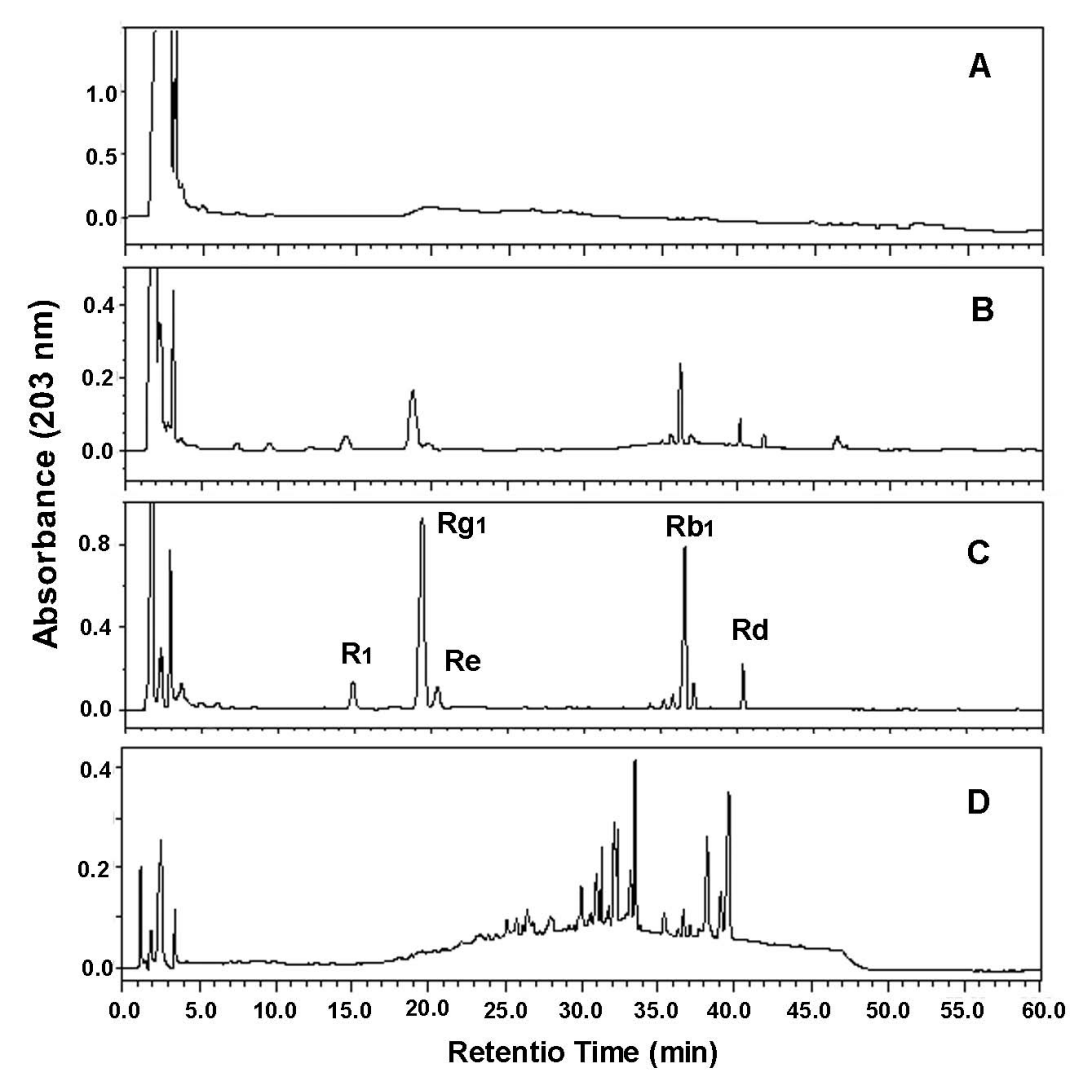
**HPLC profiles of the seed (A), six-month-old root (B), adult root (C) and seed MeOH extract (D) of *Panax notoginseng***.

Results of HPLC analyses indicated that saponins were undetectable in *P. notoginseng *seed, even though the amount of seed sample was 10 times as much as that of seedling root. We obtained just 0.3 g MeOH extract from 100 g of fresh seed with the yield being 0.3%; yield was about 0.83% in dried seed while it may reach 9.25% and 11.74% of total saponin in dried two-year-old and three-year-old roots respectively [11]. Furthermore, HPLC analysis indicated that Rg_1_, Rb_1_, Rd, Re and R_1 _(the main constituents in the root) were not detected in the crude seed extract whereas some other peaks appeared in the HPLC chromatogram, suggesting that the chemical composition of the seed was different from that of root (Figure 1). Further comprehensive chemical studies are required to determine the constituents of seed extract.

### Saponin accumulation in the root

**Figure 2:**
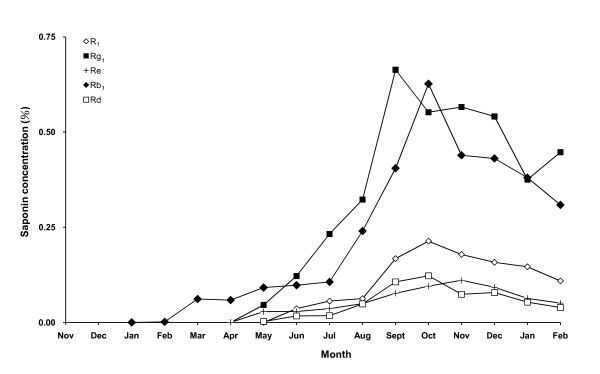
**Time courses of saponin accumulation in *Panax notoginseng *seed and seedling root**. The seeds were collected in November and stored in wet sand. They began to germinate the following January. The seedlings were then allowed to grow for one year in soil nursery bed.

After germination, Rb_1 _first appeared in the seedling root in February. Rg_1_, Re, and Rd began to appear in the fourth month of germination (the following May). After one month, all these five saponins were detected in the root. Then, with the growth of seedling, the saponin contents increased rapidly in the root. The accumulation showed a time-dependent increment of saponin contents. Rg_1 _concentration reached its maximum in September (eight months after germination). Rb_1_, Rd and R_1 _reached maximum in October whereas Re did in November. Later, as winter came, concentrations of all these five saponins started to decline significantly (Figure 2, Table 3).

## Discussion

Dammarene-type triterpenoid saponins are main secondary metabolites of *Panax notoginseng*. The present study demonstrates a temporal and spatial distribution of saponins during the germination process and the growth of young plants. Our results show that ginsenosides Rg_1_, Re, Rb_1_, Rd and notoginsenoside R_1 _were not detected in *P. notoginseng *seed. The formation of saponins in root is a gradual process. The synthesis and accumulation of saponins began after germination and continued with the growth of seedling. Saponin synthetases were activated after seed began to germinate. In young roots, saponin constituents formed and accumulated mainly between July and October, the most vigorous period of growth. This periodic change is, as in adult plant, closely related to the growth pattern of *Panax notoginseng*; the formation and accumulation of saponins were affected by seasons [12]. As a plant grows up, more and more saponins accumulate in the root. Our previous work revealed that, in a 3-year-old root, the concentrations of Rg_1_, Rb_1_, Rd, Re and R_1 _reached 4.11%, 4.12%, 0.82%, 0.83% and 1.14% respectively [11]. All these findings suggest that saponins may not serve as the nutrient storage in the seed. The protective functions of saponins in other plants are reported [13,14]. The role of this kind of secondary metabolites in *P. notoginseng *requires further investigation.

## Conclusion

The accumulation of saponins showed a time-dependent increase after germination of *P. notoginseng*.

## Competing interests

The authors declare that they have no competing interests.

## Authors' contributions

DW, YJZ and CRY designed the study. DW, HTZ and KKC carried out the cultivation. DW and MX performed the chemical analyses. DW, YJZ and CRY wrote the manuscript. HTZ and KKC cultivated and collected the samples. CRY coordinated the study. All authors read and approved the final version of the manuscript.
